# Evaluation of the immunogenic properties of the recombinant *Histophilus somni* outer membrane protein 40 kDa (rOMP40)

**DOI:** 10.1186/s12917-022-03515-x

**Published:** 2022-11-18

**Authors:** Joanna Bajzert, Katarzyna Szydłowska, Paulina Jawor, Adrianna Wawrzyniak, Maciej Pisarek, Tadeusz Stefaniak

**Affiliations:** Department of Immunology, Pathophysiology and Veterinary Preventive Medicine, Wrocław University of Environmental and Life Sciences, C.K. Norwida 31 Str, 50-375 Wrocław, Poland

**Keywords:** Recombinant OMP 40 kDa protein, Antibody cross-reactivity, *Histophilus somni*

## Abstract

**Background:**

Gram-negative bacterial infections are a serious problem in beef and dairy cattle. Bacterial outer membrane proteins (OMPs) play a pivotal role in cellular survival and the host-bacterium interaction. *Histophilus somni* OMP40 was identified as a porin with homology between its N-terminal amino acid sequence and the sequences of porins of other gram-negative bacteria The aim of this study was to produce recombinant *H. somni* OMP40 (rOMP40), optimize its production and evaluate its immunogenic properties in calves. The cross-reactivity of anti-rOMP40 antibodies were also checked.

**Results:**

The highest overexpression of rOMP40 was demonstrated by *Escherichia coli* C41 using the autoinduction process. Double immunization of calves (20 μg rOMP40 per animal) induced a significant increase of anti-rOMP40 antibodies in the IgG_1_ (*P* ≤ 0.01) and IgG_2_ (P ≤ 0.01, after first immunization only) subclasses, but not IgM. ELISA revealed increased reactivity of the IgG against surface antigens of *E. coli* and *Pasteurella multocida* after the second immunization (*P* < 0.01). Cross reactivity of anti-rOMP40 antibodies with ~ 40 kDa antigens of most common gram-negative pathogens was shown by Western blotting.

**Conclusion:**

Immunization with *H. somni* rOMP40 induced a humoral response in cattle with broad cross-reactivity with similar antigens of other species of *Pasteurellaceae* and *Enterobacteriaceae* families and the delayed-type hypersensitivity reaction. The obtained results encourage further study to evaluate the protective effect of the produced protein as a subunit vaccine in cattle.

**Supplementary Information:**

The online version contains supplementary material available at 10.1186/s12917-022-03515-x.

## Background

Gram-negative bacterial infections are a serious problem in beef and dairy cattle. The greatest losses are caused by pathogenic members belonging to the *Pasteurellaceae* family, including *Histophilus*, *Pasteurella* and *Mannheimia* strains [1], and to the *Enterobacteriaceae* family, including *Escherichia* and *Salmonella* strains [2]*.*

The bacterial outer membrane (OM), which plays a pivotal role in cell survival and the host-bacterium interaction, includes a variety of proteins [3]. Conserved OM proteins (OMPs) have become crucial immunogenic targets, which are able to induce cellular mechanisms of the host defence involving the antigen, including the release of cytokines that activate macrophages to kill the pathogen [4]. The *H. somni* OM is composed of a wide range of proteins, the most abundant of which is designed as the major outer membrane protein (MOMP) and called OMP 40 kDa (OMP40) [5]. It has been shown that the molecular mass of the MOMP varied from 43 to 33 kDa; however, most of the *H. somni* strains have a protein about 40 kDa. The characteristics of the MOMP could be one of the markers of virulence. The molecular mass of the MOMP and its reactivity with selected anti-MOMP monoclonal antibodies (mAbs) could be used for preliminary grouping of *H. somni* strains [6]. It was suggested that several epitopes of the *H. somni* MOMP are surface-exposed, and some of these epitopes are conserved and others variable among strains [3]. In the secondary structure model of *H. somni* MOMP, putative 16 transmembrane β-strands and eight loops of variable sequence and length facing the exterior side of the OM have been identified. The comparison of the MOMP derived from six unrelated strains of *H. somni* to that of strain 8025 revealed identities in the nucleotide sequences in 65.4–99.9% and in amino acid sequences in 62.4–99.7%. The lowest sequence similarity was shown for the MOMP of strain 129Pt isolated from the prepuce of healthy bovine carriers [5].

It was shown that sera obtained from cattle, swine, dogs, horses and poultry vaccinated with whole *H. somni* cells revealed a strong immune response against selected *H. somni* antigens, among others those against MOMP [7]. MOMP appeared immunogenic in rabbits and calves. The cross-reactivity of rabbit antiserum with whole-cell antigen of different/selective *H. somni* strains revealed the reaction with proteins with a molecular mass of ~ 40 kDa [6]. Similar results were obtained in immunoblot analysis of reactivity of two anti-MOMP mAbs (no. 59–8-2; 34–2-1) [8].

*H. somni* OMP40 was identified as a porin with homology between its N-terminal amino acid sequence and the sequences of porins of other gram-negative bacteria [3, 5]. The porins showing high similarity to *H. somni* OMP40 are *Actinobacillus actinomycetemcomitans* OMP39, *P. multocida* OmpH, *H. influenzae* P2 [5] and *E. coli* OmpC [3].

The aim of this study was to produce recombinant *H. somni* OMP40, optimize the harvest conditions and evaluate its immunogenic properties in calves. The cross-reactivity of anti-rOMP40 antibodies was checked by evaluation of antigen recognition in selected gram-negative bacteria, while their influence on bacterial phagocytosis and growth inhibition was also determined.

## Results

### Expression and purification of recombinant protein

The comparison of amino acid sequences of the obtained construct with sequences available in the BLAST database revealed a 99% sequence conformity for rOMP40 originating from *H. somni* (the test sequence was compared to sequences of accession numbers BBC21771.1). Analysis of the amino acid sequence revealed a difference in the amino acid incorporated at positions 2, 304 and 328 compared to the reference sequence (G was added; E changed to R; E changed to G, respectively).

**Fig. 1:**
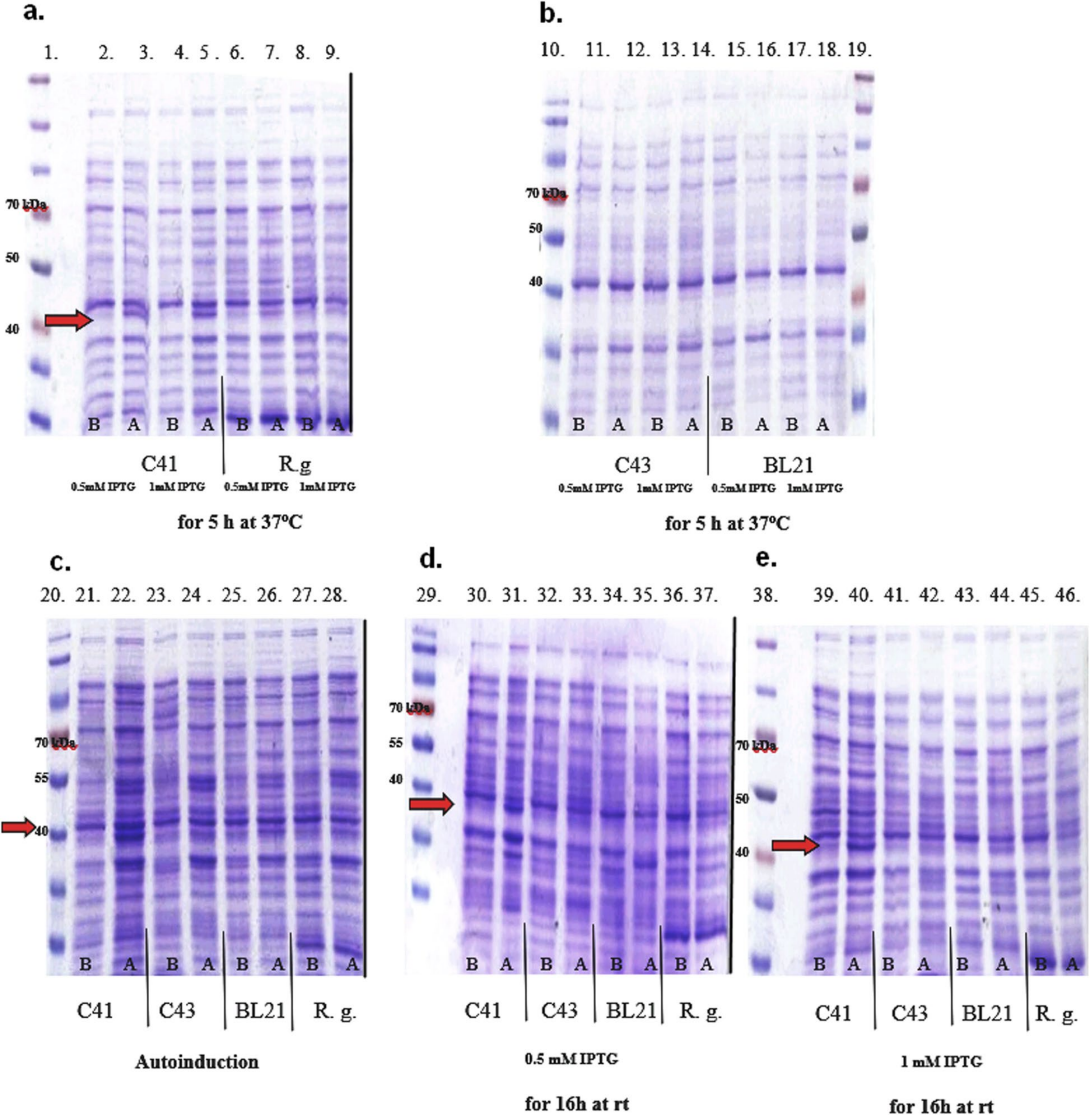
*H. somni* rOMP40 expression using various expression strains and culture conditions. Legend: SDS-PAGE pattern of selected *E. coli* expression strains (3 × 10^7^ cfu/lane) producing rOMP40 under different cultured conditions. a. - e. subsequent stained gels; A. Cells after induction of protein expression; B. Cells before induction of protein expression. C41 [C41 (DE3)], C43 [C43 (DE3)], BL21 [BL21 (DE3)], R.g. [Rosetta-gami (DE3) pLysS] – *E. coli* expression cells; gel a.- the rOMP40 production was induced using 0.5 or 1 mM IPTG and incubated later for 5 h at 37 °C; C41 and R.g. strains were used; gel b.- the rOMP40 production was induced using 0.5 or 1 mM IPTG and incubated later for 5 h at 37 °C; C43 and BL21 strains were used; gel c.- the rOMP40 was produced in the autoinduction process; C41, C43, BL21 and R.g. strains were used; gel d.- the rOMP40 production was induced using 0.5 mM IPTG and later incubated for 16 h at room temperature; C41, C43, BL21 and R.g. strains were used; gel e.- the rOMP40 production was induced using 1 mM IPTG and later incubated for 16 h at room temperature;; C41, C43, BL21 and R.g. strains were used. Samples were added to individual lanes 1–46. Lanes 1, 19, and 38: Spectra Multicolour Broad Range Protein Ladder (5 μL/lane); Lanes 20 and 29: Page Ruler Prestained Protein Ladder (5 μL/lane). Arrow shows 40 kDa antigen

rOMP40 was produced using different culture conditions and expression cells. The majority of host *E. coli* strains, such as BL21, C43 and R. gami, did not express or expressed a very low amount of rOMP40. The highest overexpression of protein was demonstrated by *E. coli* C41 strain in all tested culture conditions (Fig. 1; lines: 3; 5; 22; 31; 40).

The presence of protein in the distinct cell fractions of the *E. coli* C41 strain induced at 1 mM IPTG for 16 h at room temperature and during the autoinduction process was checked. It was elicited that both the cytoplasmic soluble and insoluble fractions contained the target protein. The amount of the protein in the soluble fraction seemed to be comparable for both means of its production, whereas in the insoluble fraction, a higher level of protein was produced by the autoinduction process. The medium fraction did not contain rOMP40. The periplasmic fraction turned out to contain a small amount of rOMP40.

**Fig. 2:**
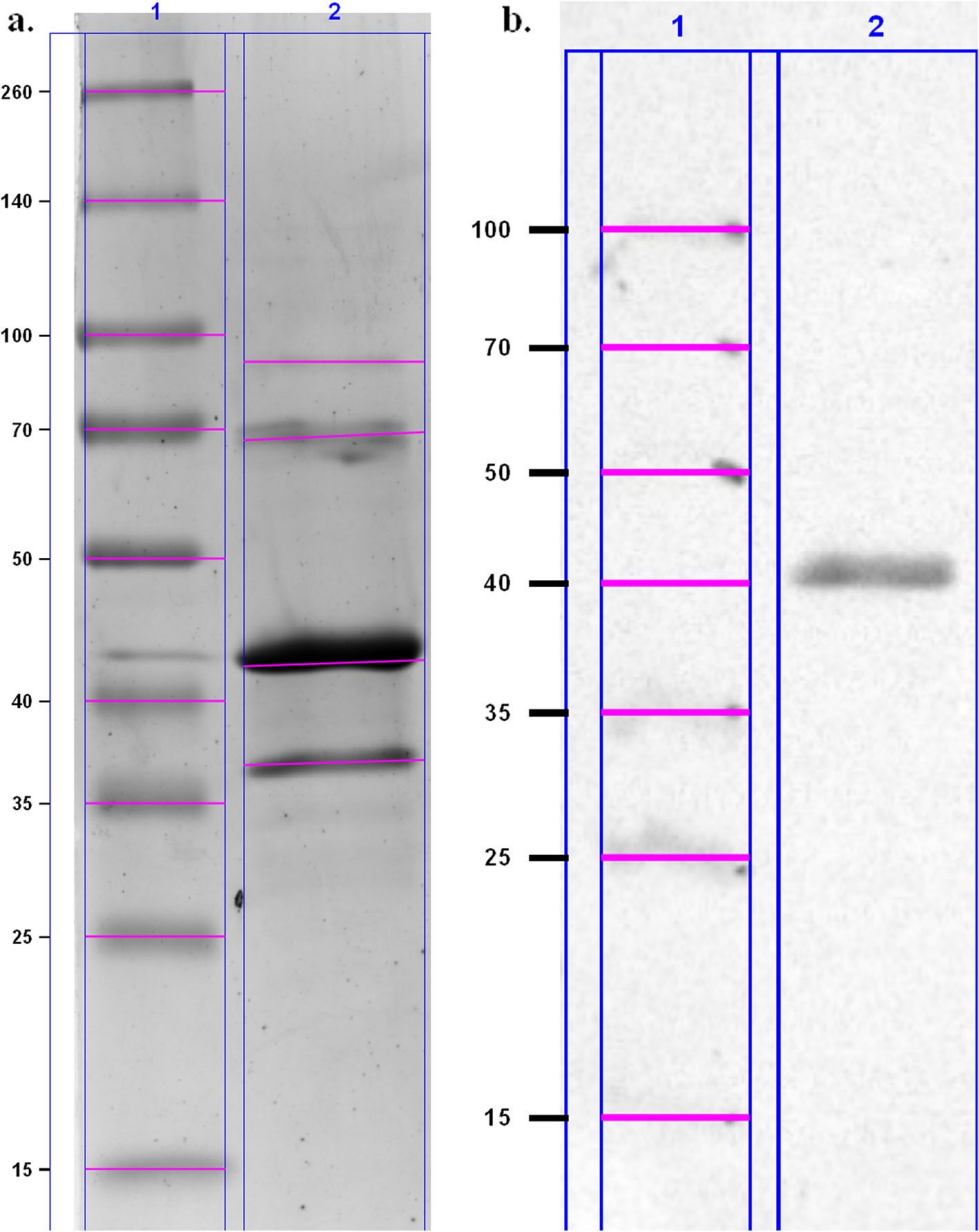
The evaluation of *H. somni* rOMP40 purity. Legend: SDS-PAGE analysis of rOMP40 antigen (**a**) and western blot analysis of the reaction of rOMP40 antigen (3 μL/lane) with mouse monoclonal anti-HisTag antibodies (**b**). Legend: Lane 1: Spectra Multicolor Broad Range Protein Ladder (Fermentas) (3 μL/lane); Lane 2: rOMP40 produced using autoinduction process by *E. coli* C41 (DE3) strain

The protein production showed that the yield achieved by the autoinduction process exceeded 836 μg/L of culture, while using IPTG, the yield was only 392 μg/L (protein was obtained from the soluble and insoluble fractions). For immunization, the protein produced using the autoinduction process by *E. coli* C41 (DE3) strain was used. SDS-PAGE analysis of affinity-purified and dialysed protein showed presence of four protein with molecular mass 90.3 kDa (very weak bands); 68.7 kDa; 42.4 kDa (the most intensive bands); 36.9 kDa (Fig. 2a). Immunoblotting analysis of protein with anti-HisTag antibodies revealed a high purity (Fig. 2b). The molecular mass calculated using Image Lab™ software was 42.1 kDa.

### Determination of reactivity of bovine serum IgG_1_ and IgG_2_ subclasses and IgM class antibody against *H. somni* rOMP40 by ELISA

**Fig. 3:**
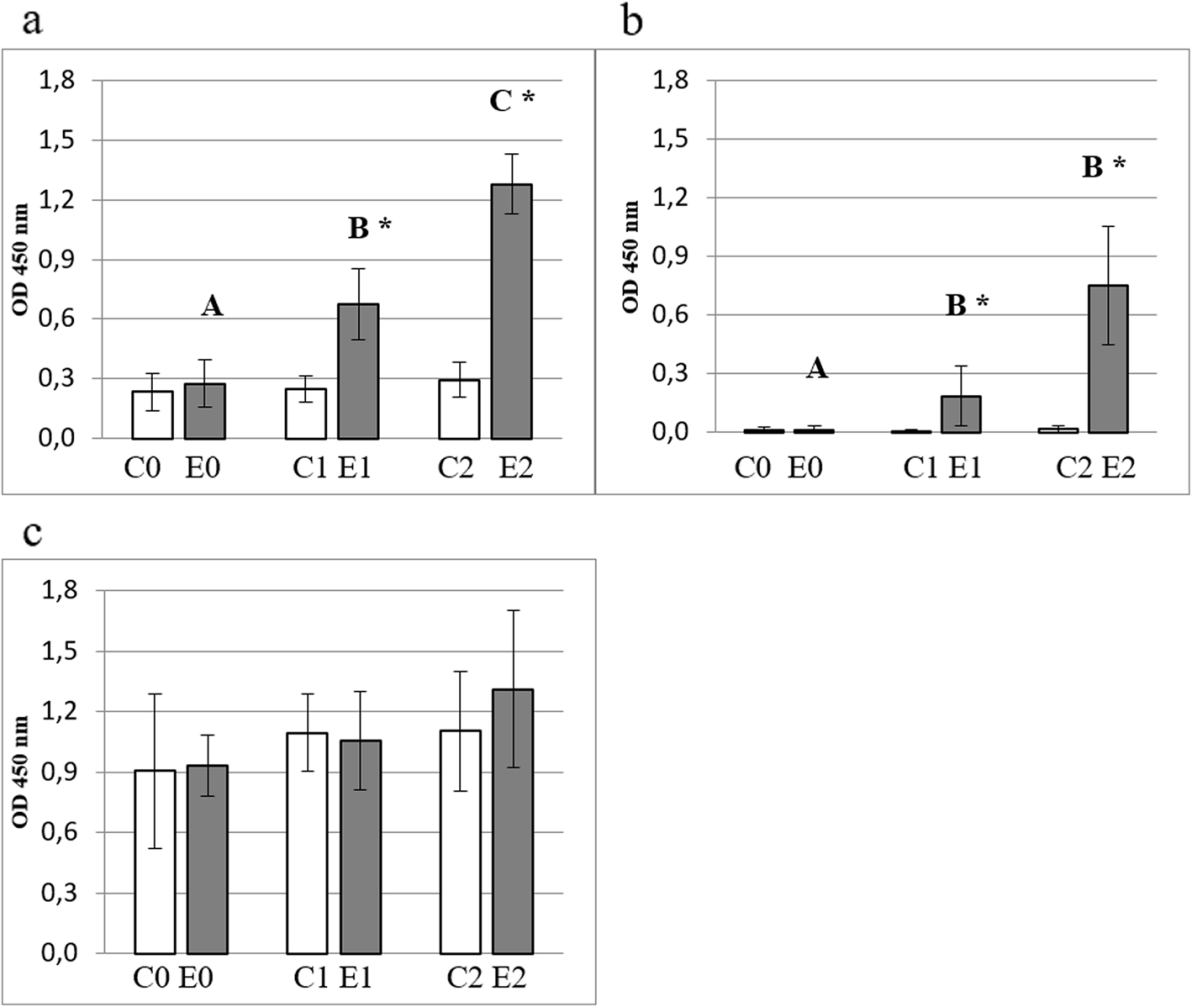
Reactivity of IgG_1_ (**a**), IgG_2_ (**b**) and IgM (**c**) antibodies against rOMP40 antigen in ELISA. Legend: Figures show the determination of reactivity of IgG_1_ (**3.a**), IgG_2_ (**3.b**) and IgM (**3.c**) antibodies against rOMP40 antigen. The blood samples were obtained before the first immunization/injection (E0, C0), 3 weeks after first immunization/injection (E1; C1) and 2 weeks after the second immunization/injection (E2; C2). Individual study groups are labelled with C0, C1, and C2 (white bars) and E0, E1, and E2 (gray bars). Significant differences within individual study groups (C0-C2; E0-E2) are labelled with different uppercase letters (**A**-**C**) for *P* ≤ 0.01. Significant differences between study groups in respective dates (C0-E0; C1-E1; C2-E2) are labelled with an asterisk (*) for *P* ≤ 0.01. ELISA plates were coated with *H. somni* rOMP40 antigen (**3.a**-**3.c**). Sera were diluted 1:100

Except for the IgG_2_ subclass antibody in the control group, in all serum samples anti- *H. somni* rOMP40 antibodies were detected (Fig. 3.a–c). Immunization induced a significant increase in IgG_1_ in the experimental group after the first and second immunizations (*P* ≤ 0.01). The difference in IgG_1_ reactivity between groups was significant in the second and third sampling (Fig. 3.a; *P* ≤ 0.01). A significant increase of IgG_2_ reactivity after immunization was detected in the experimental group in the second and third samplings, compared to the first sampling (*P* ≤ 0.01). Results in this group were significantly different compared to the control (in the second and third samplings; *P* ≤ 0.01; Fig. 3.b). In the case of the IgM antibodies, the analysis of results revealed no significant differences in reactivity of antibodies (Fig. 3.c).

### Determination of reactivity of bovine serum IgG antibodies against whole bacteria cells

**Fig. 4:**
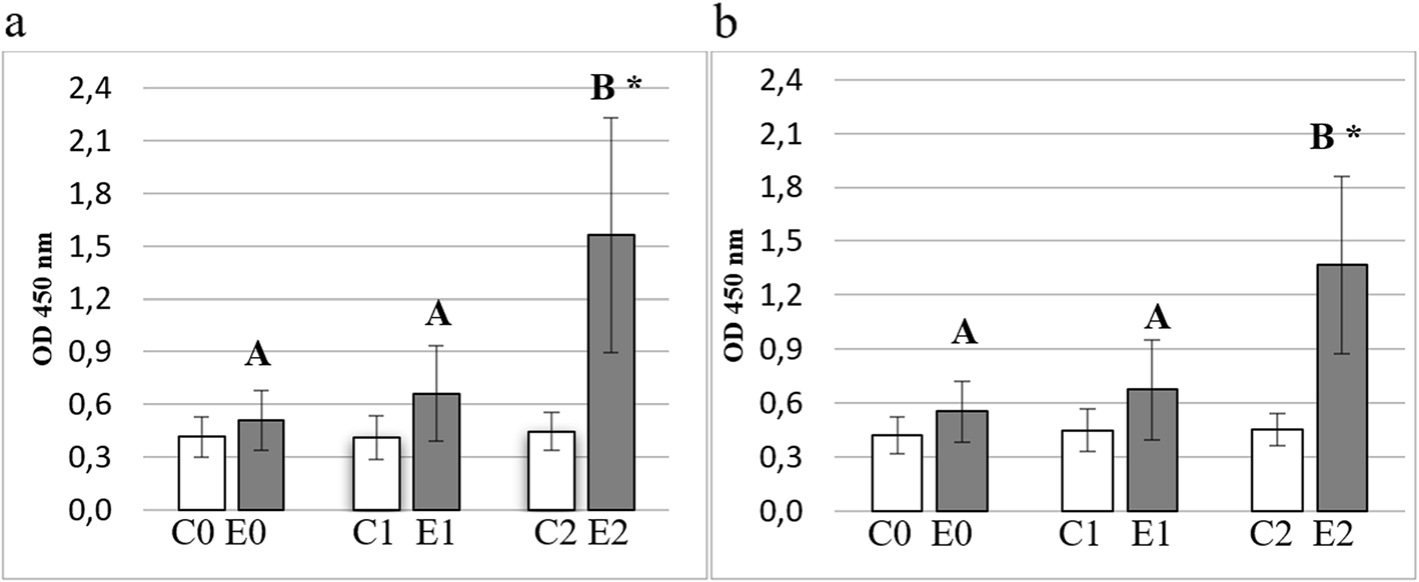
Reactivity of IgG antibodies against whole cells *E.coli* (**a**) and *P. multocida* (**b**) in ELISA. Legend: Figures show the reactivity of IgG antibodies against whole bacterial cells of *E.coli* (**4.a**) and *P. multocida* (**4.b**). The blood samples were obtained before the first immunization/injection (E0, C0), 3 weeks after the first immunization/injection (E1; C1) and 2 weeks after the second immunization/injection (E2; C2). Individual study groups are labelled with C0, C1, and C2 (white bars) and E0, E1, and E2 (gray bars). Significant differences within individual study groups (C0-C2; E0-E2) are labelled with different uppercase letters (**A**-**B**) for *P* ≤ 0.01. Significant differences between study groups in respective dates (C0-E0; C1-E1; C2-E2) are labelled with an asterisk (*) for *P* ≤ 0.01. ELISA plates were coated with *E. coli* (**4.a**) and *P. multocida* (**4.b**) whole cells. Sera were diluted 1:3000

Specific antibodies were detected in the serum of the control and experimental groups. After the second immunization a significant increase in the reactivity of the IgG antibodies specific against antigens expressed on the surface of bacterial cells (*E. coli* and *P. multocida)* was observed (Fig. 4.a, b; *P* ≤ 0.01). Results in experimental group in last sampling were significantly different compared to the control (*P* ≤ 0.01).

**Fig. 5:**
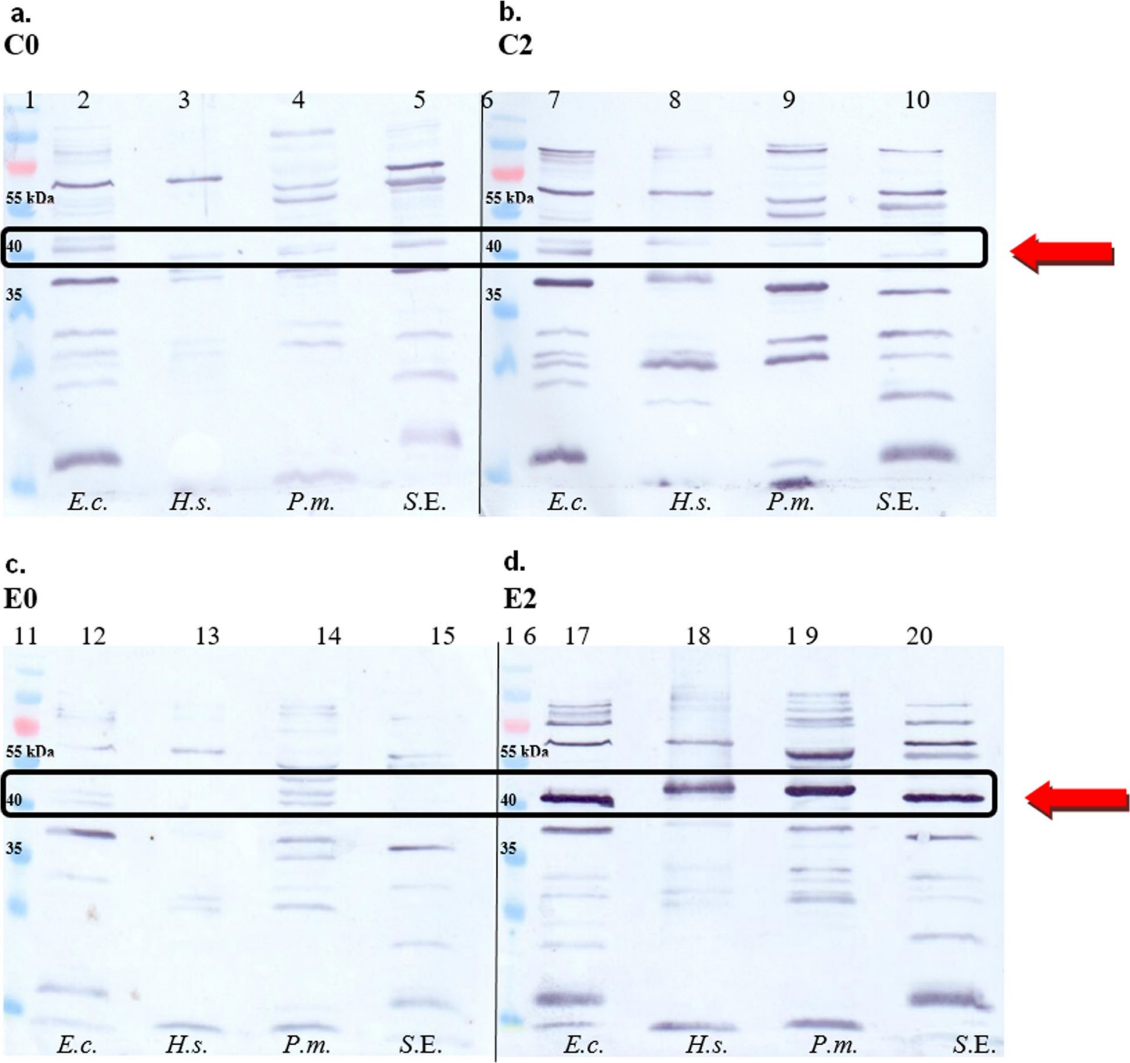
Immunoblotting analysis of the bacterial antigens with pooled calf serum obtained before and after immunization. Legend: a. - d. subsequent blots; Lanes 1, 6, 11, and 16: Page Ruler Prestained Protein Ladder (5 μL/lane); *E.c*.- *Escherichia coli* (3 × 10^7^/lane); *H.s.*- *Histophilus somni* (3 × 10^7^/lane); *P.m*.- *Pasteurella multocida* (3 × 10^7^/lane); S.E.- *Salmonella* Enteritidis (3 × 10^7^/lane); blot a.- membrane was incubated with pool of serum obtained from control calves before first injection (C0); blot b.- membrane was incubated with pool of serum obtained from control calves 2 weeks after second injection (C2); blot c.- membrane was incubated with pool of serum obtained from experimental calves before first immunization (E0); blot d.- membrane was incubated with pool of serum obtained from experimental calves 2 weeks after the second immunization (E2). As a secondary antibodies anti-bovine IgG conjugated with HRP were used. Serum pools were diluted 1:20. Arrows and frames show ~ 40 kDa antigens

In Western blotting (Fig. 5), the intensive cross-reactivity of IgG antibodies of all animals with selected proteins of gram-negative pathogens was observed. Pooled serum samples obtained from the experimental (E2) and control groups (C2) revealed a reaction with antigens of molecular mass ~ 88 kDa, ~ 60 kDa, ~ 37 kDa, ~ 30 kDa and 25 kDa. A strong reaction with antigens with a molecular mass of ~ 40 kDa was observed only with the pooled serum obtained 2 weeks after second immunization (Fig. 5.d; E2). The strongly recognised proteins possessed the molecular mass: 42.2 kDa for *H. somni*, 41.4 kDa for *E. coli*, 43.9 kDa for *P. multocida*, and 40 kDa for *Salmonella Enteritidis*.

The comparison of amino acid sequences of the obtained protein with sequences available in the BLAST database revealed a 34% sequence conformity for porin originating from *P. multocida*, a 29% sequence conformity for porin OmpC originating from *E. coli*, and a 23% sequence conformity for porin originating from *S. enteritidis* (the test sequence was compared to sequences of accession numbers OIQ13297.1, WP_053890044.1, and MJY20408.1, respectively).

### Antibody-dependent bacterial growth inhibition

**Fig. 6:**
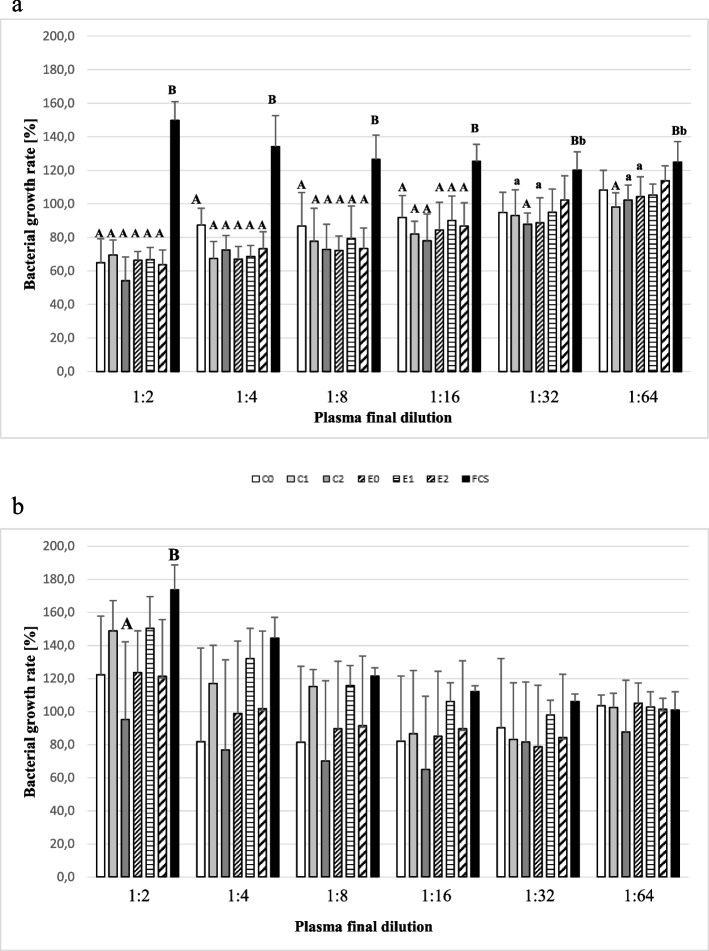
Antibody-dependent *E. coli* (**a**) and *P. multocida* (**b**) growth inhibition. Legend: The pooled serum samples obtained from calves immunized with *H. somni* rOMP40 (E) and the control group (C) were tested for their activity against *E. coli* (**6.a**) and *P. multocida* (**6.b**) growth. As a positive control, FCS was used. All pools of serum and FCS were diluted at a two-fold dilution series in the range of 1:2–1:64. Bacteria (*E. coli* and *P. multocida*) were preincubated with inactivated pool of serum obtained from control/experimental calves: C0/E0- before first injection/immunization (white bar/ thin diagonal strips bar); C1/E1–3 weeks after the first injection/immunization (light gray bar/ transverse strips bar); C2/E2–2 weeks after the second injection/immunization (dark gray bar/ fat diagonal strips bar) and FCS- foetal calf serum (black bar). Measurements of bacterial growth inhibition were repeated five times. Bacterial growth inhibition was calculated using the following equation/formula: Bacterial growth rate [%] = OD_600nm_ for bacteria preincubated with selected serum pool or FCS: OD_600nm_ for bacteria preincubated with PBS * 100%. Significant differences between study groups are labelled with different lowercase letters (a-b) for *P* ≤ 0.05 and different uppercase letters (**A**-**B**) for *P* ≤ 0.01

The incubation of *E. coli* with pooled serum samples (Fig. 6.a) obtained from the experimental group (E0, E1, E2) and the control group (C0, C1, C2) diluted in the range of 1:2 to 1:16 significantly (*P* ≤ 0.01) decreased the bacterial growth rate compared to growth with FCS. In dilution of 1:2, the bacterial growth rate did not exceed 80%. At pooled serum dilution 1:32, a significant decrease of bacterial growth was detected in groups C2 (*P* ≤ 0.01), C1 and E0 (*P* ≤ 0.05) compared to growth with FCS. At pooled serum dilution 1:64, a significant decrease of bacterial growth was detected in groups C1 (*P* ≤ 0.01), C2 and E0 (*P* ≤ 0.05) compared to growth with FCS.

The incubation of *P. multocida* with serum pools (Fig. 6.b) obtained from the experimental group, as well as from the control group, did not decrease the bacterial growth rate. Addition of the pooled serum (except C2) diluted by 1:2 increased bacterial growth to the level observed for the positive control (FSC). Only the addition of C2 pooled serum (diluted 1:2) caused a significantly lower bacterial growth rate (P ≤ 0.01) as compared to the growth with FCS.

### The influence of post immunization opsonins on phagocytosis by bovine granulocytes

Intensive phagocytosis of bacteria (*E. coli* and *P. multocida)* by granulocytes was observed (Fig. S1). No significant differences in phagocytosis were shown for both strains of bacteria after their incubation with post immunization serum pools (normal and inactivated; E2) compared to the control pooled sera (normal and inactivated; C2), as well as to FCS. Surprisingly, the most intensive phagocytosis was observed for bacteria incubated without serum additive (MFI = 1448.5 ± 752.2 for *E. coli* and MFI = 1554.1 ± 446.7 for *P. multocida*). However, it was not significantly different compared to serum-treated bacteria.

### Double skin fold thickness

Before intradermal injection the thickness was not different between groups (mean ± SD 4.6 ± 0.9 and 5.3 ± 0.9 mm, control and experimental respectively). The significant increase in the skinfold thickness was observed in experimental groups 24 h (8.9 ± 2.2 vs 5.8 ± 1.0 mm) and 48 h (7.3 ± 1.3 vs 5.5 ± 0.8 mm) after antigen injection (*P* ≤ 0.05) comparing to control group. The thickness of the skin was not significantly different on 72 h (6.3 ± 0.8 vs. 5.4 ± 0.8 mm) experimental vs control respectively.

## Discussion

*H. somni* causes significant losses due to multisystemic diseases of cattle [9]. It was observed that hyperimmune goat serum developed by immunization with crude OMP of *H. somni* induced cross-reactivity with whole cell antigens of examined strains of *Pasteurellaceae* and *Enterobacteriaceae* families; one of the strongest reactions was observed with ~ 40 kDa antigens [own observation, unpublished]. These results encouraged us to obtain recombinant *H. somni* OMP40 antigen and identify its immunogenic properties in cattle. Furthermore, the effects of anti-rOMP40 antibodies on phagocytosis and bacterial growth were evaluated.

Because the production of recombinant proteins circumvents the costs of purifying native antigens from whole bacteria and there is little [5] evidence about optimising the production of rOMP40 derived from *H. somni*, we made an attempt to obtain this protein using genetic engineering methods. Many systems may be available for the expression of recombinant proteins, but only a few combinations of vector, host strain and culture conditions allow the efficient production of active proteins. In our study, we searched for the combination that gave the highest yield of rOMP40. Out of four different *E. coli* expression strains only one, C41, overproduced rOMP40. This strain was derived from Origami and designed for expressing toxic transmembrane proteins [5, 10] such as OMP40. We found that production of rOMP40 using the autoinduction system was the most efficient, probably because autoinduction is designed for high-level protein expression with the pET system. One of the benefits associated with this kind of induction is the fact that we do not need to monitor the culture and add inducers during cell growth. These systems often increase cell mass and target protein yield [11].

In the second step of the study, we verified the protein localisation in the bacterial cell. We observed that *H. somni* rOMP40 could be isolated from both soluble and insoluble cytoplasmic fractions. In many applications, it is desirable to express proteins in their soluble, active form. For this reason, we chose the pET system, which enhances solubility by adding a signal sequence for translocation of the protein into the periplasmic space. We prolonged IPTG induction at a low temperature (16 h at room temperature) [12], and we used special *E. coli* strains, such as R. gami*,* which are permissive for the formation of disulphide bonds in the cytoplasm [13, 14]. However, those changes did not achieve the desired results. Membrane-associated proteins, such as OMP40, can precipitate as inclusion bodies, which we showed by purifying a high amount of rOMP40 from the insoluble fraction. Probably, in our case, formation of inclusion bodies could be very advantageous, because toxic protein may not inhibit cell growth when present in an inactive form. The obtained recombinant protein was insoluble in buffers, probably because of its membrane origin and hydrophobic moiety, but it remained soluble when 10% glycerol was added.

In the next step of our study, the immunogenicity of *H. somni* rOMP 40 kDa in cattle was examined. OMPs such as 17.5 kDa [15], 37 kDa [16], ~ 40 kDa (OMP40/MOMP) [3, 5, 6], 39 kDa [17], 40 kDa [17–19], 76 kDa [20] and 78 kDa [18, 21] have been studied as *H. somni* potential virulence factors so far. In some cases [16, 17, 21], antibodies against these proteins showed cross-reactivity with homologous proteins of other gram-negative bacteria.

In this study, we obtained protein whose nucleotide sequence coincides with the sequence of the rMOMP (major outer membrane protein) produced by Khan et al. [5]. We would like to emphasize that the protein obtained is not the protein described by Corbeil et al. [17, 22, 23] and Googlewski et al. [18, 24] as a immunodominant OMP 40 kDa. Corbeil et al. [17, 23] arbitrarily categorised *H. somni* OMPs as p41 (MOMP at about 41 kDa), p40 (immunodominant OMP at about 40 kDa), and p39 (OMP which reacts with mAb 3G9). The comparison of some selective sequence data and molecular masses of MOMP (p41) from selective *H. somni* strains revealed their high diversity in the range of 32.914 kDa (strain 129Pt) to 40.439 kDa (strain 540) [5]. Tagawa et al. [6] showed that the molecular mass of the MOMP can vary from 33 kDa to 43 kDa, and all proteins have common epitopes reacting with selected mAbs. The molecular mass of rOMP40 obtained in this study, calculated using ExPASy tools, was 41.7 kDa (without the last eight aa, sequence LEHHHHHH), using Image Lab was 42.1 kDa (whole aa sequence). In immunoblotting analysis, the molecular mass of the *H. somni* (strain CAMP 6280) protein strongly recognised by anti-rOMP40 calves serum was 42.2 kDa. Tagawa et al. [3, 6] showed that MOMP with molecular mass about 40 kDa are usually identified in pathogenic *H. somni* strains, especially in those isolated from a case of bovine TEME (thromboembolic meningoencephalitis), pneumonia and less in the case of myocarditis and abortion (Group 1). A few asymptomatic carrier strains have a truncated MOMP of about 33 kDa [6].

Corbeil et al. [17, 25] and Gogolewski et al. [24] showed that the bovine immune response (in the IgG class of antibodies) against MOMP (p41) during *H. somni* infection is very weak and probably induced production of IgE antibodies [22]. Corbeil [23] suggested that MOMP may not be a good candidate to create a subunit vaccine, not only due to its weak immunological reactivity with serum from infected convalescent cattle, but also due to its antigenic variability. However, Tagawa et al. [8] demonstrated that several epitopes of the MOMP are exposed on the bacterial surface, and enhancement of the immune response against MOMP by immunization seems to be highly justified.

In the present study, rOMP40 was used for immunization of cattle. We would like to emphasise that the immunogenicity of the native MOMP in bacterial cells may differ from that purified by Tagawa et al. [3] and Corbeil et al. [17], which were probably denatured. Recombinant *H. somni* OMP40 produced by using standard genetic engineering methods could have not only liner but also conformation epitopes, which could play a key role during antibody reaction with the bacterial surface. We showed that double immunization with doses of 20 μg of rOMP40 (emulsified with adjuvant) was sufficient to induce a humoral immune response in calves. Tagawa et al. [3] showed that the MOMP of *H. somni* (strain 8025; purified by ion-exchange and molecular-sieve chromatographies, then ethanol precipitated) is immunogenic to rabbits and calves. For experiments with rabbits, four subcutaneous immunization with doses of 200 μg (10-times higher a dose than used in this study) emulsified with Freund’s adjuvant complete (FAC; first immunization)/Freund’s adjuvant incomplete (FAI; other immunizations) were used. In case of calves, three subcutaneous immunization with doses of 500 μg (25-times higher a dose than used in this study) emulsified with FAI was done. Immunisation induced the antibody response against the purified MOMP in IgG class. The reactivity of the anti-MOMP antibody increased markedly during the immunization.

In conducted experiments, the rapid rise of specific anti-rOMP40 antibody of IgG_1_ and IgG_2_ subclasses indicates its high immunogenicity. Lack of increasing reactivity of antibodies of the IgM class was observed, and similar pattern was observed in calves when animals were vaccinated with *H. somni* rHsp60 [26]. This could be the effect of the former primary immune response against highly conservative proteins, which are widely spread in *Procaryota* [27]. As shown in this study, IgG_1_ was the predominant subclass of antibody formed in response to the subcutaneous immunization of calves with rOMP40. Individual subclasses of antibodies are characterized by different biological activities and functions. In bovines, IgG_1_ is the principal immunoglobulin for passive immunization of the calf [28]. IgG_2_ antibodies appear more homogeneous than IgG_1_ antibodies and occur in high concentrations in bovine serum [28], which is crucial in protection against pyogenic infections [29, 30]. The role of IgG_2_ in resistance to extracellular bacterial infections is probably due to the fact that it is more efficient as an opsonin than IgG_1_ and also activates the bovine complement cascade [31, 32]. Because some gram-negative bacteria, such as *P. multocida*, *P. haemolytica* [33], *Salmonella* Minnesota [34], *S. typhimurium*, and *E. coli* (only serum-sensitive strain) [35] are susceptible to killing by complement, the presence of anti-OMP40 IgG_2_ antibody in the blood of animals seems to be advantageous during infection.

In the next step of the experiment, it was demonstrated that calves *H. somni* rOMP40 sera showed intensive cross-reactivity with ~ 40 kDa antigens of other gram-negative bacteria (immunoblotting and ELISA). This cross reactivity of anti-rOMP40 antibodies with proteins form other gram-negative bacteria should be evaluated in future if it would provide protectivity. Until now, the cross-reactivity of the anti-MOMP antibodies has been shown only with different strains of *H. somni* [6], own observation, unpublished]. In our experiments, we demonstrated that anti-rOMP40 antibodies recognised antigens with molecular mass about 40 ± 2 kDa from members of the *Pasteurellacea*e and *Enterobacteriaceae* families. In this way, they could increase the recognition of bacteria during the infection. It is noteworthy that the antibodies against porin proteins of *H. influenzae* [36] and *Neisseria gonorhoeae* [37], *S. typhimurium* [38] showed bactericidal and protective effects.

In this study, the response to intradermal injection of antigen in the cellular response test was measured. Cell-mediated immune response (CMIR) plays a pivotal role in protection against intracellular pathogens. In vivo, it is manifested as a delayed-type hypersensitivity (DTH) [39]. It was shown that many different antigen-adjuvant combinations induced DTH [40]. Skin DTH has two phases. First, there is a sensitization (after first contact with antigen), then after subsequent exposure to the same antigen, the effector phase is observed. The inflammatory reaction peaks 24–72 h after antigen exposure [39]. In this study, for the first time, the DTH reaction for *H. somni* rOMP40 protein emulsified with Emulsigen was shown. The response in the experimental group was the strongest 24 h and 48 h after injection. The observed peak reactions at 24–48 h corresponds with results obtained by Hernández et al. [40] and Kelley et al. [41]. Until the DTH reaction in cattle after *H. somni*, antigen injections have been shown only for *H. somni* Hsp60 [26].

In the last part of the experiment, the role of *H. somni* rOMP40 antibodies in bacterial growth inhibition and phagocytosis was examined. *H. somni* is bacterium that requires demanding growth conditions and grows very slowly; therefore, all experiments investigating bacterial growth inhibition and phagocytosis were carried out using *Pasteurella multocida* as a related member of the *Pasteurellacea*e family. Instead of the strong reaction of the obtained antiserum with bacterial antigens expressed on their surface in the ELISA test, we did not find marked inhibition of bacterial growth after their preincubation with post-immunization serum. In the literature, it has been shown that antibodies against the OMPs inhibit proliferation of different gram-negative bacteria, such as *P. multocida* [42, 43], *E. coli*, *K. pneumoniae* [44] and *Vibrio* spp. [45]. Uneco et al. [46] observed high susceptibility to killing of *E. coli* and *H. influenzae* by normal, nonactivated bovine serum but *P. multocida* and *M. hemolytica* were serum resistant. In this study, inhibition of *E. coli* growth caused by bacterial agglutination was observed. However, there was no difference in intensity of *E. coli* growth rate between all tested sera (control and experimental). This means that, in all serum samples, the level of antibodies reacting with antigens expressed on the surface of *E. coli* was already high, and anti-rOMP40 antibodies did not impact on stronger bacteria recognition. Because all tested serum samples were inactivated, the role of the complement was excluded. In the case of *P. multocida*, it was observed that the addition of tested sera to the bacterial culture led to bacterial growth at a level comparable with FCS supplemented culture. Collins [47] showed that the addition of 10% FCS to heart infusion broth greatly increased the *P. multocida* growth rate. Probably, the *P. multocida* strain used in this study was resistant to the obtained antiserum. Ataei Kachooei et al. [48] showed that *P. multocida* B:2 was highly resistant to positive serum (containing high levels of IgG and IgM antibodies, obtained from immunized calves) and also highly resistant to complement activity in normal fresh calf serum.

Phagocytosis is the process by which macrophages, neutrophils and dendritic cells internalise particulate material [49]. Phagocytes must recognise a large number of different particles, and this recognition is achieved thanks to a variety of discrete receptors. Receptors on the plasma membrane of phagocytes can be divided into non-opsonic (as lectin-like recognition molecules, directly recognise molecules on the pathogen’s surface) or opsonic receptors (Fc receptors, complement receptors (CRs)) [39, 50]. Phagocytosis becomes essential for microbial elimination [50]. Many pathogens are known to modulate phagocytosis [49]. In the experiments conducted, we checked if bacterial preincubation with serum containing anti-rOMP40 antibodies and complement increased bacterial phagocytosis. Intensive phagocytosis of bacteria (*E. coli* and *P. multocida)* by granulocytes was observed; however, we did not observe an increase of bacterial immunophagocytosis after their preincubation with calf pooled serum harvested 2 weeks after the second immunisation. What’s more, there was no difference between normal and inactivated sera. The results obtained may suggest that a pivotal role in bacterial recognition is played by pattern recognition receptors that detect molecules typical of the pathogens. Additionally, OMP40 antigen expressed on the bacterial surface was probably recognised by predominant anti-rOMP40 IgG_1_ antibodies. IgG_1_ subclass *H. somni* rOMP40 antibodies were able to agglutinate the bacteria, but probably not improved the immunophagocytosis process. There is a question whether the dye used for bacterial staining, which is a non-nucleic acid labelling reagent, did not disturb the recognition by Fc receptors and CRs.

## Conclusion

In this study, we produced *H. somni* rOMP40 and showed that rOMP40 induced a humoral response in cattle with broad cross-reactivity with similar antigens of other species of *Pasteurellaceae* and *Enterobacteriaceae* families. The DTH reaction shows that cell-mediated immune response was also induced. Therefore this protein may to be interesting as a potential component of a subunit vaccine. The confirmation or dismissal of our assumption needs to be evaluated in experimentally infected animals and in large groups of animals in field conditions.

## Materials and methods

### Bacterial strains

*E. coli* reference strain 0111:K58(B4):H No. 27 originated from the Polish Collection of Microorganisms Wrocław; *H. somni* reference strain CAMP 6280 originated from Czech Collection of Microorganism, Brno. *P. multocida* strain was isolated from bovine and *Salmonella enterica* spp. *enterica* ser., Enteritidis strain isolated was from poultry. These both strains originated from the collection of the Department of Epizootiology and Clinic of Birds and Exotic Animals, Wrocław University of Environmental and Life Science, Poland.

### Animals

The study was conducted on a commercial dairy farm, with 170 cows and a milk yield of 6700 kg per lactation. Fifteen calves either Holstein-Friesian (*n* = 9, four in control group) or Holstein-Friesian-Simmental crossbreeds (*n* = 6, three in control group) were enrolled in the study. Calves were between 31 and 70 days of age at the beginning of the study and were kept in group pens (control and experimental animals mixed in pens with other calves on the farm) with straw bedding (six per each pen). Calves were fed with milk replacer twice daily with 3 L until approximately 75 days of age and had ad libitum access to hay and prestarter. Eight animals were enrolled in the experimental group (mean ± SD 54 ± 15 days of age) and seven to the control group (mean ± SD 51 ± 14 days of age).

All experiments were carried out in line with the recommendations set forth in the Act on Animal Protection (Journal of Laws of 1997, No. 111, Item 724) and were approved by the Local Ethics Committee in Wrocław (Local Ethics Committee Resolution No. 25/2012 from 19.03.2012).

### Antigen preparation

Recombinant OMP 40 kDa from *Histophilus somni* (reference strain CAMP 6280) was obtained using standard genetic engineering methods. Amplification of the rOMP40 gene (accession number LC160262.1) was performed using the PCR protocol. In the PCR reaction, an approximately ~ 1143 bp fragment was amplified using specific primers, whose sequence encoding sites are recognised by the restriction enzymes NcoI and XhoI (primer sequences: sense 5’GCCCATGGGCAAAAAGACATTAGTAGC3’; antisense 5’CGCTCGAGGAAGTAAACACGT AAAC3’). To avoid the reading frame shift on the N-terminus of protein sequence three extra nucleotides encoding glycine were added. The PCR mix consisted of 1 × buffer, 500 μM of dNTP mix, 400 nM of each primer, 2% DMSO, 2.5 units of Accu Taq Polymerase (Sigma-Aldrich, St. Louis, MO, USA) and 1–5 μL of purified DNA (isolated from *H. somni* CAMP 6280) in a final volume of 50 μL. PCR was performed as follows: after an initial denaturation step of 10 minutes at 94 °C, a set of 30 cycles was run, each consisting of 15 seconds at 94 °C, 30 seconds of annealing at 52 °C and 90 seconds at 68 °C, followed by a final extension of 10 min at 68 °C. An amplified DNA fragment was cloned into a multiple cloning site in the pET 22b(+) plasmid (Novagen, Madison, USA) using specific restriction enzymes (NcoI and XhoI, Thermo Fisher Scientific, Waltham, USA). The nucleotide sequence of the cloned genes was analysed by automatic sequencing across the cloning junction, using the universal primer T7 (Genomed S.A., Warsaw, Poland). Recombinant plasmids were transformed into distinct *E. coli* expression cells. The DNA and amino acid sequences of *H. somni* rOMP40 were deposited in the NCBI (National Center of Biotechnology Information) database; accession number OP562169. The amino acid sequence of the *H. somni* OMP40 protein was compared with sequences of other gram-negative bacteria (like *P. multocida* sequence accession number OIQ13297.1; *E. coli* WP_053890044.1; *S. enteritidis* MJY20408.1) using BLAST database.

### Protein expression and purification

Optimisation of the rOMP40 production was performed using an autoinduction process or induction using isopropyl-*β*-d-1-thiogalactopyranoside (IPTG). For protein production, different *E. coli* expression cells were used as follows: C41 (DE3), C43 (DE3) (Lucigen, Middleton, USA), Rosetta-gami (DE3) pLysS and BL21 (DE3) (Novagen).

Protein production using an autoinduction process was performed as previously described [51]. When protein production was induced using IPTG (Thermo Fisher Scientific), the transformed bacteria were cultured overnight in 100 mL of lysogeny broth (LB) medium containing ampicillin (100 μg/mL Polfa Tarchomin S.A., Warsaw, Poland; for *E. coli* C41, C43 and BL21) and chloramphenicol (35 μg/mL Sigma-Aldrich; for *E. coli* Rosetta-gami pLysS). This preinoculum was added to 1.9 L of fresh LB medium containing appropriate antibiotics in a fermenter (MoBiTec, Goettingen, Germany). The bacteria were grown to OD_600_ = 0.6 and then induced using 0.5 or 1 mM IPTG and incubated for 5 h at 37 °C or 16 h at room temperature at 250 rpm. After incubation, the cultures were centrifuged at 2000×g for 20 minutes and the pellets stored at − 20 °C.

The cytoplasmic insoluble and soluble fractions and periplasmic fraction of bacterial cells were isolated following the instructions from the Manual pET System Novagen 10th Edition [52]. Bacterial cell fractions, expression cells (before and after induction; 3 × 10^7^ cfu per lane) and OMP40 protein were separated by SDS-PAGE (12% gel). Gels were stained using Coomassie Brillinat Blue R-250 (Sigma-Aldrich). rOMP40 identification using immunoblotting was performed as previously described [51]. Gel and membrane visualisation was made using ChemiDoc Touch Instruments (BioRad). The molecular weights of proteins were estimated using Image Lab™ software by comparison with standard protein marker.

Proteins were purified using a Ni-NTA column as previously described [51]. For the purpose of maintaining the solubility of protein, 10% glycerol (Sigma-Aldrich) was added. The eluates were dialysed against phosphate buffered saline (PBS) (pH = 7.4) containing 10% glycerol.

### Immunization of calves with rOMP40

All eight calves in the experimental group were injected twice, subcutaneously, in the neck with 20 μg of the emulsified antigen. Booster doses were given after 21 days. rOMP40 dissolved in saline was emulsified with Emulsigen-D (MVP Laboratories, Omaha, USA); the final volume of the vaccine’s dose per animal was 1 mL. One dose of vaccine consists of 20 μg rOMP40 + 200 μL Emulsigen-D (20% v/v) + saline added to the volume 1000 μL. The control group (seven animals) was injected with a 0.8 mL saline emulsified with the same adjuvant (0.2 mL; 20% v/v). Blood samples were obtained:

1. immediately before first immunization/injection (E0 for the experimental group; C0 for the control group),
2. three weeks after first immunization/injection (E1; C1), then second immunization/injection was done,
3. two weeks after second immunization/injection (E2; C2).
  The acquired blood sera were collected and stored at − 20 °C.

### Double skin fold thickness

A skin test was performed in eight calves of the experimental group and in seven control animals 2 weeks after second immunization. In each animal, a cross-shaped pattern was shaved into the fur on the right side of the neck. The skin was cleaned and disinfected with 70% alcohol. All animals were injected intradermally with 0.2 mL rOMP40 (25 μg/mL). The skinfold thickness was measured with a calliper immediately before the antigen injection and 24, 48 and 72 h after.

### Detection of bovine serum antibody against *H. somni* rOMP40 and whole bacterial cells by ELISA

Blood serum obtained from the experimental group (E0, E1, E2) and from the control group (C0, C1, C2) was examined for the presence of IgG_1_, IgG_2_ and IgM antibodies against purified rOMP40 and for the IgG cross-reactive antibodies against antigens expressed on the surface of whole bacteria (*E. coli* and *P. multocida*). ELISA was performed as previously described [51] with some modification.

Microplates (Nunc Maxisorp; Thermo Fisher Scientific) were coated either with rOMP40 (3 μg/mL in 0.05 M carbonate buffer pH = 9.6, 50 μL per well) or with whole *E. coli* and *P. multocida* cells (2 × 10^8^ cfu/mL in PBS buffer pH = 7.4; 100 μL per well). The plates were blocked using PBS buffer containing 0.1% Tween 20 (Sigma-Aldrich; 200 μL per well). Serum samples were diluted by 1:100 (to detect IgG_1_, IgG_2_ and IgM antibodies against rOMP40) or 1:3000 (to detect IgG antibodies against whole bacterial cells) with PBS containing 0.05% Tween 20. Dilution of HRPO conjugates were 1: 100000 for sheep anti-bovine IgG_1_; 1:120000 for sheep anti-bovine IgG_2_; 1: 25000 for sheep anti-bovine IgM (BioRad, Hercules, USA); and 1:80000 for goat anti-bovine IgG (Sigma-Aldrich). The plates were then incubated with supersensitive TMB substrate (Sigma-Aldrich), in the dark at room temperature for 20 minutes for the rOMP40 antibody and 25 minutes for the whole bacteria antibody. All samples were assayed in duplicate.

### Examination of the reactivity of calves serum antibody with bacterial antigens by immunoblotting

Immunoblotting was performed as previously described [51] with some modification. Whole bacterial cell antigens (3 × 10^7^ cfu per lane) of gram-negative bacteria, representative of the *Pasteurellaceae* (*H. somni, P. multocida*) and *Enterobacteriaceae* (*E. coli*, *S. enteritidis*) families were separated by SDS-PAGE (12%). The membranes were incubated with a pool of calves serum (four pool was prepared: before immunisation sera from E0 and C0 and after the second immunization sera from E2 and C2; dilution 1:20) at 4 °C for 18 h. As the second antibody, goat anti-bovine IgG HRPO conjugate (Sigma-Aldrich; dilution 1:3000) was applied. The results of western blot documentation was made with ChemiTouch Instruments (BioRad). The molecular weights of proteins were estimated using Image Lab™ software (version 5.2.1; BioRad) by comparison with standard protein markers (PageRuler™ Prestained Protein Ladder, Thermo Fisher Scientific).

### Antibody-dependent bacterial growth inhibition

Bacteria (*Escherichia coli*, *Pasteurella multocida*) were grown in LB medium at 37 °C, at 250 rpm to an optimal density (OD_600nm_) of 0.220. They were then diluted 1:1660 in LB medium. A total of 10 μL of bacterial suspension was added to 1490 μL physiological saline and mixed vigorously. The mixture was added (25 μL/well; ~ 20 cfu/well) to a two-fold serial dilution of serum pools (25 μL/well; C0,C1,C2; E0,E1,E2; final dilution rates of 1:2–1:64) to sterile 96-well plates. Bacteria were also incubated with foetal calf serum (FCS, Fetal Bovine Serum, Gibco Thermo Fisher Scientific; positive control) and with PBS buffer (negative control). All serum pools and FCS (final dilution rates of 1:2–1:64) were inactivated by incubation for 20 minutes at 56 °C. Plates were incubated in a mini-rocker shaker at 37 °C for 90 minutes at 70 rpm. Then, 150 μL of LB medium was added, and the plates were incubated in a mini-rocker shaker at 37 °C for the next 18 h at 70 rpm. After incubation, the bacteria culture in each well was mixed by vigorous pipetting. The OD_600nm_ was measured (BioTek uQuant microplate reader). To exclude bacterial contamination of reagents, all samples (LB medium, saline, serum pools and FCS) were incubated on the plates without adding bacteria. All samples were assayed in duplicate. The experiment was repeated five times.

Bacterial growth inhibition was calculated using the following formula:

Bacterial growth rate [%] = OD_600nm_ for bacteria preincubated with selected serum pool or FCS: OD_600nm_ for bacteria preincubated with PBS * 100%.

### Phagocytosis assay

From adult, healthy, non-pregnant cow venous blood was taken using heparin (5 U/mL) as an anticoagulant. The blood sample (3 mL) was centrifuged at 800 x g for 10 minutes, then serum and half of the centrifuged cells were removed. The polymorphonuclear leucocyte (PMN) fraction of the blood cells was isolated from the remaining cells using 0.84% ammonium chloride solution (incubation for 7 minutes at 37 °C). After incubation, the cells were centrifuged at 300 x g for 7 minutes. Cells were washed twice using PBS (pH = 7.4). Simultaneously, bacteria (*E. coli* and *P. multocida*) were prepared. Bacteria were grown in LB medium for 16 h at 37 °C at 250 rpm. They were then preincubated with bovine intact or inactivated serum pools (E2 and C2) or intact or inactivated FSC for 15 minutes at 37 °C at 70 rpm. The final dilution of serum pools and FSC was 1:5. After preincubation, bacteria cells were washed twice using a PBS buffer (centrifuged for 10 minutes at 2500 x g) and resuspended in 1 mL of physiological saline. Next, 1 μL of BacLight bacterial stain (Thermo Fisher Scientific) was added to the samples and incubated for 15 minutes at room temperature. Then, the bacteria were washed twice using a PBS buffer. Stained bacteria were incubated for 10 minutes at 37 °C with granulocytes (ratio 100:1; the final concentration of bacteria was 3 × 10^7^ cfu, granulocyte concentration was 3 × 10^5^ cells) in a final volume of 300 μL of RPMI medium. As a control of spontaneous phagocytosis, bacteria and granulocytes were incubated for 10 minutes at 4 °C. Then, the cells were centrifuged at 1000 x g for 10 minutes at 4 °C at the highest centrifugal acceleration (1.55 min) and deceleration (1.47 min) and cell pellets were resuspended in 300 μL of PBS buffer. Samples were analysed by flow cytometry. A flow cytometer (FACSCalibur, BD Biosciences,USA) equipped with a laser emitting at 488 nm was used. Fluorescence was collected in the green-fluorescence channels. To determine the optimal compensation settings, the appropriate controls, including unstained bacteria, stained bacteria and granulocytes were analysed. The level of the mean fluorescence intensity (MFI) was measured. All samples were analysed in duplicate. The bacterial phagocytosis rate was calculated using the following formula: MFI of bacterial phagocytosis at 37 °C − MFI of bacterial phagocytosis at 4 °C.

The experiment was repeated nine times.

### Statistics

Statistical analyses were carried out on Microsoft Excel spreadsheets and STATISTICA software v.12.5 (StatSoft Inc., Tulsa, OK, USA). Mean absorbance levels from ELISA and antibody-dependent bacterial growth inhibition tests, as well as mean MFI from the phagocytosis assay, are presented in bar graphs, including standard deviations (SDs). Statistical analyses were carried out on the means of duplicate measurements performed for each determination of serum levels in the group (acceptable CV = 10%). Data were checked for normality using the Lilliefors test. The results of the absorbance in the ELISA test had abnormal distributions; therefore, logarithmic transformation (log10) was performed before analysis. Values were tested using one way ANOVA and post hoc comparisons were made using Tukey’s HSD test. Two levels of statistical significance were assumed (*P* ≤ 0.05 and *P* ≤ 0.01). Results of skin thickness from the tests were compared with the Mann-Whitney U test.

## Supplementary Information

**Additional file 1: Figure S1.** The influence of post vaccination opsonins on phagocytosis by bovine granulocytes. Legend: The influence of the pooled serum samples obtained from rOMP40 immunized calves (E2) and the control group (C2) were tested. Bacteria (*E. coli*- white bar and *P. multocida*- dark gray bar) were preincubated with non- or inactivated serum pools (final dilution 1:5): B- only stained bacteria; FCS- foetal calf serum; C2- pool of serum obtained from control calves 2 weeks after the second injection; E2- pool of serum obtained from experimental calves 2 weeks after the second immunization; in- inactivated serum. The bacteria were stained and incubated with granulocytes (ratio 100:1). The level of the mean fluorescence intensity (MFI) was measured. The bacterial phagocytosis rate was calculated using the following equation/formula: MFI of bacterial phagocytosis at 37°C- MFI of bacterial phagocytosis at 4°C. All experiments were repeated nine times.

## Data Availability

The data sets supporting our results are included in the article. Raw data are available upon request to corresponding author (JB: joanna.bajzert@upwr.edu.pl). The DNA and amino acid sequences of recombinant *Histophilus somni* outer membrane protein 40 kDa (*H. somni* rOMP40) were deposited in the NCBI (National Center of Biotechnology Information) database; accession number OP562169.

## Abbreviations

OMPs: Outer membrane proteins
rOMP40: Recombinant outer membrane proteins 40 kDa
MOMP: Major outer membrane protein
mAbs: Monoclonal antibodies
E: Experimental group
C: Control group
C41 (DE3), C43 (DE3), BL21 (DE3), Rosetta-gami (DE3) pLysS: *Escherichia coli* expression cells
IPTG: Isopropyl-*β*-d-1-thiogalactopyranoside
DTH: Delayed-type hypersensitivity
