# Supplementary figures and images for: Evaluation of the immunogenic properties of the recombinant Histophilus somni outer membrane protein 40 kDa (rOMP40)
Source: BMC Vet Res. 2022 Nov 18;18:409. doi: 10.1186/s12917-022-03515-x (PMC9673221; doi:10.1186/s12917-022-03515-x)

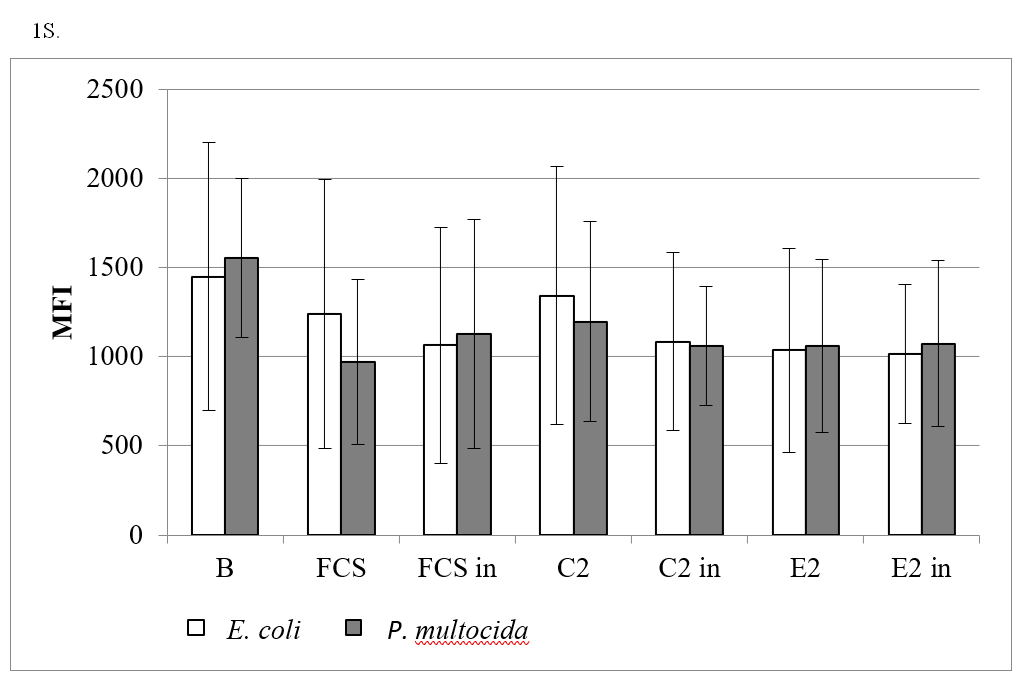

Supplement: Supplementary file 1 — Additional file 1: Figure S1. The influence of post vaccination opsonins on phagocytosis by bovine granulocytes. Legend: The influence of the pooled serum samples obtained from rOMP40 immunized calves (E2) and the control group (C2) were tested. Bacteria (E. coli- white bar and P. multocida- dark gray bar) were preincubated with non- or inactivated serum pools (final dilution 1:5): B- only stained bacteria; FCS- foetal calf serum; C2- pool of serum obtained from control calves 2 weeks after the second injection; E2- pool of serum obtained from experimental calves 2 weeks after the second immunization; in- inactivated serum. The bacteria were stained and incubated with granulocytes (ratio 100:1). The level of the mean fluorescence intensity (MFI) was measured. The bacterial phagocytosis rate was calculated using the following equation/formula: MFI of bacterial phagocytosis at 37°C- MFI of bacterial phagocytosis at 4°C. All experiments were repeated nine times. [file 12917_2022_3515_MOESM1_ESM.tif]
